# A Novel Approach towards Medical Entity Recognition in Chinese Clinical Text

**DOI:** 10.1155/2017/4898963

**Published:** 2017-07-05

**Authors:** Jun Liang, Xuemei Xian, Xiaojun He, Meifang Xu, Sheng Dai, Jun'yi Xin, Jie Xu, Jian Yu, Jianbo Lei

**Affiliations:** ^1^Second Affiliated Hospital Zhejiang University School of Medicine, Hangzhou, Zhejiang Province 310000, China; ^2^Sir Run Run Shaw Hospital Zhejiang University School of Medicine, Hangzhou, Zhejiang Province 310000, China; ^3^Zhejiang University International Hospital, Hangzhou, Zhejiang Province 310000, China; ^4^National Center for Advancing Translation Sciences, National Institutes of Health, 9800 Medical Center Drive, Building C, Room 312, Rockville, MD 20850, USA; ^5^Hangzhou Medical College, Hangzhou, Zhejiang Province 310000, China; ^6^Peking University Center for Medical Informatics Center, Beijing 100191, China; ^7^Southwest Medical University, Luzhou, Sichuan Province 646000, China

## Abstract

Medical entity recognition, a basic task in the language processing of clinical data, has been extensively studied in analyzing admission notes in alphabetic languages such as English. However, much less work has been done on nonstructural texts that are written in Chinese, or in the setting of differentiation of Chinese drug names between traditional Chinese medicine and Western medicine. Here, we propose a novel cascade-type Chinese medication entity recognition approach that aims at integrating the sentence category classifier from a support vector machine and the conditional random field-based medication entity recognition. We hypothesized that this approach could avoid the side effects of abundant negative samples and improve the performance of the named entity recognition from admission notes written in Chinese. Therefore, we applied this approach to a test set of 324 Chinese-written admission notes with manual annotation by medical experts. Our data demonstrated that this approach had a score of 94.2% in precision, 92.8% in recall, and 93.5% in F-measure for the recognition of traditional Chinese medicine drug names and 91.2% in precision, 92.6% in recall, and 91.7% F-measure for the recognition of Western medicine drug names. The differences in F-measure were significant compared with those in the baseline systems.

## 1. Introduction

The existing natural languages are mainly divided into alphabetic and logographic languages. Logographic languages contain written characters representing words or phrases, and the best known logographic language is Chinese, while alphabetic languages are made of written characters representing sounds or sound combinations rather than concepts [1]. Electronic health records (EHRs) have been widely adopted worldwide. In the health care reform in China, National Health and Family Planning Commission of PRC has considered the nationwide application of EHRs one of its priorities and launched a set of modestly interoperable Chinese EHR Template Guides (CEHRTG) in 2010 [2–4]. In 2013, Chinese Hospital Information Management Association conducted an investigation on EHRs using data from the healthcare information systems in China. The study that involved 1004 Chinese medical institutions has shown that electronic medical records (EMRs) are prevalently applied in these hospitals (77.8%) and a portion of these hospitals have incorporated the EMR systems into regional or state-level EHRs [5]. A large number of studies on data mining and knowledge discovery have been devoted to the clinical data accumulation in EHRs. However, structured data mining methods cannot be directly used to mine EHRs stored in nonstructural texts; thus, these texts need to be preprocessed with structuration [6]. Named entity recognition (NER) in free narrative forms of clinical texts aims to recognize relevant named entities (NEs) from nonstructural clinical data and is also a basic task of medical language processing (MLP) [7]. Researches on medical entity recognition (MER) in admission notes (ANs) written in logographic languages, such as Chinese, lag behind researches on MER in ANs written in alphabetic languages, such as English. Such a gap between the two areas of research is largely due to the huge differences between alphabetic and logographic languages [8] and between modern Western medicine (WM) and traditional Chinese medicine (TCM) [9], which hinder the utilization of alphabetic research results in logographic investigation in fields of both entity recognition in general and MER in specific. In particular, with supervised algorithms based on discriminative models [10], we have to solve many urgent problems to study nonstructural Chinese admission notes involving TCM-WM combined treatment.

1. Is there a generalized approach for efficient MER for nonstructural EHRs written in a logographic language?
2. Is it feasible to selectively extract concrete medical features from free narrative clinical texts, so that supervised algorithms can fulfill MER tasks in clinical ANs written in a logographic language?
3. Among some supervised algorithms [e.g., support vector machine (SVM), conditional random field (CRF) with specific characteristic types] that are widely applied in NER, which of them are applicable for MER in ANs written in a logographic language?

Here, we proposed a new cascade-type MER approach called cascade-type Chinese medication entity recognition (CCMER) for processing admission notes in Chinese. We aimed to test CCMER in clinical texts in English (an alphabetic language) and investigate the roles of 4 feature types based on supervised algorithms [6] used for MER in nonstructural Chinese admission notes with TCM-WM combined treatment. This is the first time an MER is used under such context. Moreover, we designed and conducted a series of fine experiments using CCMER in ANs with TCM-WM combined therapy written in both Chinese and English and compared the performance of CCMER with that of three baseline systems (one based on rules, one based on simple local context features, and the other based on simplified CCMER with the filtration module for classifications of potential hot-sentence deletion). According to the soft scores of correctly recognized drug name entities, we statistically analyzed the results of CCMER and the baseline 2 system as follows:

(1) $$\begin{array}{c} \mu_{\overset{-}{CCMER}}=0.90;\;\mu_{\overset{-}{baseline\;2}}=0.87;\\ \sigma_{\overset{-}{CCMER}}=0.24;\;\sigma_{\overset{-}{baseline\;2}}=0.27. \end{array}$$

Results showed that the performance was significantly improved (*P* < 0.05). This study provides guiding and reference values for MLP of nonstructural logographic EHRs.

## 2. Related Works

Across the world, key information search in abundant nonstructural ANs through computer-based resolution was used to assist better clinical decisions as early as 1967. Depending on the purposes and contexts, key information search can be realized either by information retrieval (IR) at document level (recognition and identification of relevant AN documents from massive free text-formatted ANs) [11–13] or through finer recognition, identifying various descriptions with identical meaning in clinical texts and mapping them to the same concept. MER is a typical concept-level information extraction (IE) task and also a major and basic area in MLP research [6].

The MER of early MLP systems usually adopted rule-based methods [14]. In recent years, machine learning- (ML-) based MER methods have been gradually applied in processing clinical narrative texts. One important reason for this trend is the availability of abundant anonymous clinical ANs and introduction of libraries of annotated words as gold standards, both issued by Informatics for Integrating Biology and the Bedside (I2B2) [15]. For instance, the theme of the 3rd I2B2 contest was extraction of information about drug names, doses, usage, and duration from discharge summaries [7]; one subtheme of the 4th I2B2 contest was extraction of concepts about illness state, examination, and treatment [16]. Later, these datasets from clinical ANs inspired a number of studies on NER in English clinical notes. These NER tasks were usually transformed to sequence annotations and recognized by supervised ML algorithms, such as conditional random field and structural support vector machine (SSVM). These preliminary works revalidated the three major factors that affected the systematic performance of supervised ML algorithms based on discriminative models for NER tasks: input features of descriptive datasets, model selection for classification function, and learning process for determination of model parameters [17]. We think that “appropriate feature selection” is especially important for MLP tasks, because current techniques for parameter determination are ineffective in the context of high-redundancy and high-noise featured clinical AN texts. Studies on processing clinical texts in other alphabetic languages other than English, such as French, German, and Dutch, have been carried out as well, mapping clinical descriptions to Systematized Nomenclature of Medicine codes [18].

However, most of the studies described above are limited to clinical ANs written in alphabetic languages. Studies for NER in logographic languages are relatively few. With the wide application of EHRs in China, it is urgent to test the reliability of the secondary use of abundant Chinese clinical ANs. So far, little effort has been devoted to NER in ANs written in logographic languages, especially in Chinese. As reported, several ML-based algorithms including CRF, SVM, and maximum entropy have been used to identify symptom descriptions and pathogenic mechanisms from TCM EMRs [19]. Also, CRF has been used to identify biomedical name entities in Chinese research abstracts [20]. A parallel implementation of word segmentation and NER tasks has been proposed to replace the serial application of the two tasks, in order to improve the performances of NER in discharge summaries written in Chinese [21]. Some studies on the performances of several ML algorithms for NER in clinical WM ANs written in Chinese are highly valuable [22]. However, there is no research on an MER task in Chinese AN texts with TCM-WM combined treatment, especially on WM and TCM drug NERs, yet such texts are the most commonly used in Chinese hospitals.

Here, based on document-level IR of ANs [23], we proposed a new cascade-type MER algorithm (called CCMER) and validated it in ANs written in Chinese. We also aimed to test MER in English clinical texts (English is a representative of alphabetic languages) and investigate the roles of 4 feature types based on supervised algorithms [6] for MER in nonstructural texts of TCM-WM combined treatment in Chinese ANs. The MER we presented here is the first MER of its kind. This study might provide guiding and reference values for MLP for logographic languages in general.

## 3. Dataset and Annotation

### 3.1. Description and Definition of Drug Named Entities

Based on the standard writing formats of WM and TCM prescriptions in medical orders [24] and a combination of the specific format of medical orders in an AN dataset and the characteristics of drug named entities, we compiled a list of WM/TCM drug-related treatment events and their definitions (see Table 1) in ANs [25].

In the actual clinical environment, medical orders regarding TCM or WM may contain only WM and TCM drug named entities without any other descriptive terms of the related drug use events.

### 3.2. Annotation Rules on Word Library of Drug Named Entities

To improve the reliability of manually annotated drug names, we established detailed annotation rules based on the above basic definitions. We subdivided medications into Western medication and traditional Chinese medication and further improved TCM-related annotation rules. The name annotation rules of core drugs are listed in Table 2.

### 3.3. Dataset Description

Totally 1000 out of 120,000 CEHRTG-based ANs between January 2011 and December 2012 recorded in SAHZU were extracted randomly. After deletion of incomplete ANs, 972 ANs were kept and then anonymized before manual annotation: private information including ID, name, sex, age, and reception department was deleted. Then, two native Chinese-speaking nurses annotated the names of clinical WM and TCM drugs according to the predefined guidance. To measure the interannotator agreement (IAA), the nurses independently annotated the same 100 ANs, and a senior doctor evaluated the annotations by using arbitration on disagreements. Potential issues were identified and the predefined annotation guideline was modified when necessary. The most difficult step for concept annotation of Chinese drug names is the determination of the boundary of an expression. Using the modified annotation guideline, the nurses manually annotated drug names in the remaining 872 admission notes. Therefore, 100 notes were annotated by both nurses, and the remaining 872 ANs were evenly divided and annotated by each nurse.

Then, the total 972 ANs were divided into 2 sets: 2/3 (648 ANs) as a training set and 1/3 (324 ANs) as a test set. The feature set for a classifier's optimal performance was determined by 10-fold cross-validation using the training set. The performance was then assessed using the test set. The statistics of used data are listed in Table 3.

The 972 ANs contain 61,046 sentences, with 2739 mentioning drug names. There are totally 2599 drug named entities, including 1903 WM ones (73.2%) and 696 TCM ones (26.8%). Based on the 100 ANs annotated by both experts, the IAA was measured by kappa statistics to be 0.968, which indicates that the annotation was reliable.

## 4. Methods

### 4.1. CCMER Framework

**Figure 1:**
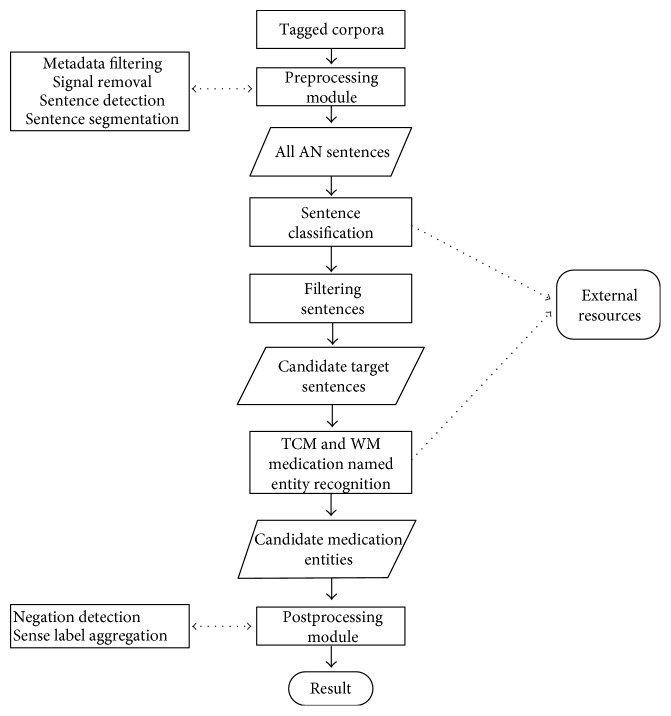
High-level architecture for the CCMER.

CCMER is a new-pipelined cascade-type framework scheme. It locates hot-text chunks based on sentence classification and recognizes drug named entities from candidate target sentences via a supervised algorithm for sequence annotation. The system structure (Figure 1) includes a preprocessing module, a filtration module for potential hot-sentence classification, a recognition module for identifying drug NER, and a postprocessing module.

### 4.2. Preprocessing Module

The preprocessing module directly runs on the original document set. Because of specific writing habits, ANs with TCM-WM combined treatment contain abundant figures, Chinese characters, and English words. So such AN documents should be normalized first so as to reconstruct the inputted sentences, which can then be processed by the standardized natural language processing (NLP) tool. For instance, numbers and Greek letters are normalized to 9 and @, respectively. Then, with the character-to-pinyin function on Microsoft Office Word 2011, we transformed all Chinese characters into pinyin. Finally, the sentence splitter from the adjusted ICTCLAS system [26] was used to segment the text into sentences. Noticeably, in this process, we did not use the stopword list [27], which is commonly used in the general NLP field. This is because owing to the transliterative characteristics of WM drug names, the Chinese names of WM drug named entities contain abundant function words, so direct deletion of function words can destroy the completeness of drug named entities. Different from English texts, Chinese texts do not have spaces between words, which further complicates the identification of word boundaries. Because of the characteristics of Chinese clinical notes (e.g., telegraphic styles, pervasive abbreviations), automatic segmentation of Chinese clinical texts into words or phrases is dramatically difficult, not to mention the recognization of details from these fragments. These early research results suggest that the general purpose segmenter trained on general Chinese texts cannot handle clinical notes well, especially on clinical terms, and may undermine its effectiveness [28]. Therefore, we did not use the approach of filtering user-defined function words, which are determined by statistical indicators (e.g., term frequency-inverse document frequency). Instead, we delivered the preprocessed texts directly into the next module.

### 4.3. Filtration Module for Classifications of Potential Hot Sentence

Though CRF-based recognizers have achieved outstanding performances in different sequence annotation tasks [29], CRF severely depends on the surrounding named entities (NEs) in local contexts and assumes that similar local contexts lead to the same judgments [28]. However, this assumption is basically untenable in the contexts of clinical ANs. For instance, a phrase in the “test results” section which refers to a specific test name might represent a drug name in the “medications” section. The same phrase under different local contexts can be identified differently. A training set with abundant misleading information like this can largely reduce the performance of the CRF-based annotator.

To delete such confusing data from the dataset, we used an SVM- based [30] user-defined sentence category classifier to filter out the irrelevant sentences that do not contain information of drug names. The potential hot-sentence classifier divides the sentences contained in ANs into two parts. One part includes sentences that may contain drug names; the other part contains the remaining sentences. Only the first part is transmitted to the subsequent CRF-based recognition module for identifying drug names, thereby reducing noise and operation time, alleviating the interference from the unbalance of negative-positive sample distribution in the corpus with the subsequent recognition module, and improving system performance. The underlying scientific hypothesis is that “every sentence in an AN belongs either to the sentence type that contains drug names or to the type that does not.” The basic assumption in the study is that a sentence is an instance in a set of hypothetical classes: “the sentences with drug names” and “the sentences without drug names.”

When the NLP system is trained by a text set composed of characters, it usually describes texts by selecting phrase characteristics first. The occurrence of a phrase is considered the most important phrase characteristics in the bag-of-words model. However, accurate segmentation of Chinese words or phrases is not easy, especially in clinical texts [31]. The accuracy in the phrase-based verbal form analysis is also reduced. Thus, we used a feature extraction function for dimension reduction, which can be interpreted as a transformation from high-dimension to low-dimension vector space. Feature extraction utilizes as feature elements the components of the sentence instance as well as pattern templates hidden in a combination of components. A pattern template includes repeatability and concurrent events. This approach leads to a more general model for character-composed cases and further reduces the probability of model overfitting.

In this feature extraction function, each sentence is described as a vector of features.

**Definition 1 .**

For a feature vector (**f**_1_, **f**_2_, **f**_3_,…, **f**_*m*_), let *n*_*ij*_ be the weight measuring the occurring frequency of feature *f*_*i*_ in sentence *s*_*j*_. The vector of sentence *s*_*j*_ is represented as  (**n**_1*j*_, **n**_2*j*_, **n**_3*j*_,…, **n**_*mj*_). Note that *n*_*ij*_ is positively related to the occurring frequency *f*_*i*_ in sentence *s*_*j*_. If feature *f*_*i*_ occurs in sentence *s*_*j*_, then, *n*_*ij*_ > 0; otherwise, *n*_*ij*_ = 0. Nevertheless, the value of *n*_*ij*_ depends on the corresponding situation.

Here, we use a 6-dimension feature vector, mainly including the features based on clinical knowledge, statistics, and linguistics separately. It is specifically defined as follows:

#### 4.3.1. In Symbol List (SF1)

The number of formal symbols (e.g., “<,” “>,” “(,” and “),” commonly used in clinical medication rules) was contained in the current sentence. If SF1 = 0, this sentence does not contain such descriptive symbols.

#### 4.3.2. In Chinese Drug Name Dictionaries (SF2)

Do the Chinese drug name terms contained in Chinese drug name dictionaries appear in the current sentence?

#### 4.3.3. In Pinyin Dictionaries (SF3)

Does the pinyin corresponding to the drug name terms contained in drug name dictionaries appear in the current sentence? The idea behind is that “An inputted Chinese character string is mapped into a voice coding sequence, which is the pinyin pronunciation or a rough approximation of the inputted string,” since the Chinese translation of English WM drug names is mainly based on transliteration. In actual clinical EHR writing, due to the wide use of the Chinese pinyin input method, when the description of drug names in ANs in Chinese characters shows writing or printing errors, the corresponding pinyin spellings might be actually correct. So different Chinese character strings with the same pronunciations are mapped to the same voice-encoded strings since they have the same pinyin spellings. This approach is essentially a feature clustering and can be used to correct many writing and printing errors.

#### 4.3.4. Positive Set of Statistical Word Features (SF4) and Negative Set of Statistical Word Features (SF5)

The features for SF4 and SF5 are represented by the sum of frequency-weighted values of statistical word features (WFs) in the current sentence. An *n*-gram-based feature extraction algorithm [32] is used to extract the 2- to 4-character keywords in the set of positive sentences (i.e., the sentences with medicine information) and in the set of negative sentences (i.e., the sentences without medicine information), respectively. To segment sentences into bigrams, this algorithm summarizes the occurring frequencies of not only the current bigram Chinese character combinations but also the adjacent ones prior to the current ones and determines which combinations occur frequently and continuously. We empirically reserve the top 30% of statistical feature phrases ranked by weight. SF4 is mined for the positive set of the sentences with medicine information and SF5 for the negative set. Finally, a total of 119 positive feature words and 313 negative feature words were extracted and reserved. Then, the feature words were weighted as per frequencies of all selected feature words appearing in the positive or negative word library. For instance, “yuyi,” a positive feature word meaning “give help or treatment to,” appears 114 times in the positive word library, while all the selected positive feature words appear 2524 times in total, so the weight of “yuyi” is 0.045.

#### 4.3.5. In Event Patterns (SF6)

Does the predefined drug use event phrase collocation template appear in the current sentence? Through observation of drug use events, we defined some phrase modes commonly used in drug use events, such as

[NUMBER + DOSEPATTERN];

[MODEPATTERN];

[FREQUENCYPATTERN];

[MODEPATTERN + CANDIDATE MEDICATION NAME];

[CANDIDATE MEDICATION NAME + FREQUENCYPATTERN].

The collocation of these phrases is a favorable indicator of drug use events.

The AN of one patient contains “Anti-inflammation treatment with provision of (Ofloxacin) Levofloxacin tablets Q.D 0.5 g for 7 continuous days in a local clinic,” which was transformed by a feature transform function into a 6-dimension feature vector:

< 2, 2, 2, 0.045, 0, 4 > ➔ MEDICATION.

The first feature indicates that the number of special characters is 2.

The second feature indicates that the candidate drug name terms appear 2 times in the current sentence.

The third feature indicates that the pinyin description corresponding to the candidate drug name terms appears 2 times in the current sentence.

The fourth and fifth features indicate that positive feature words, but not negative feature words, appear in the current sentence, with a frequency weight of positive feature words equal to 0.045.

The sixth feature indicates that the phrase templates commonly used in <MEDICATION> appear 4 times in the current sentence.

Then, we used the classifier, a stochastic gradient descent module [33], to implement the stochastic gradient descent learning model for use in a support vector machine (SVM). With the above feature sets, a linear SVM sentence classification model was trained on the training dataset and was evaluated on the test set. Finally, the sentence classifier filtered irrelevant sentences and transmitted medical sentences (the clauses containing the drug names) to the next module.

### 4.4. Recognition Module for Identifying Drug NER

Chinese ANs are special in some ways. For instance, a majority of Chinese names of WM drugs are actually the transliteration from foreign words, so an extra disambiguation of word segmentation is needed, which is much more complicated than the automatic word segmentation of general Chinese named entities. Existing common tools and methods for Chinese word segmentation thus cannot be directly applied to word segmentation of clinical ANs, which necessitates the customization for the medical field. Therefore, we did not conduct word segmentation and part-of-speech analysis commonly used in text processing. We used selected Chinese characters as the basic annotation units instead, because Chinese characters are the most basic sentence-composing units and also contain semantics.

The annotation of drug named entities can be transformed into a sequence annotation task:

(2) $$\begin{array}{c} {AN}_{x}=x_{1},x_{2},x_{3},\ldots ,x_{n}, \end{array}$$

where *x*_*i*_ is a Chinese character.

The objective is to construct an annotator *p* that can accurately annotate a reliable label sequence *y* = *p* (*x*) for a Chinese character sequence *x*, where

(3) $$\begin{array}{c} y=y_{1},y_{2},y_{3},\ldots ,y_{n},\;y_{n}\in \left\{B,I,O\right\}. \end{array}$$

The actual annotation task is finished by the CRF-based supervised ML sequence annotator, which was trained by an annotated corpus, while the training set is composed of a sequence pair (*x*, *y*). CRF is widely applied in MLP of English ANs [34] with outstanding performance. Here, we used CRF++, a tool which provides CRF-targeted efficient implementation [35] and uses L-BFGS for optimization.

In this module, we use a feature set containing 5 types of features (see Table 4).

#### 4.4.1. The Local Context Feature (F1)

F1 is composed of 2 dimensions: texts and pronunciation. The ANs with TCM-WM combined treatment contain relatively simple short narrations (e.g., short subsentences) and abbreviations of technical terms. Usually, the average length of a Chinese phrase is 2 Chinese characters [36]. Thus, we tried a Chinese character-based *n*-gram pattern as the basic unit, *n* ∈ (1, 2, 3). Relevant literatures show that the context window size (CWS) in NER tasks should not be preset to be too large [37]. Analysis of ANs shows that the fragment extent for acquisition of text features or the context window should not be too large; otherwise, more noise will be introduced, which reduces the annotation accuracy. Thus, we set the CWS < 4. For instance, C_*i*−1_C_*i*_ is treated as 2-gram, including the current and the previous Chinese characters.

#### 4.4.2. Feature of Drug Name Dictionary (F2)

F2 has two dimensions including texts and pronunciation, namely, the Chinese characters in the current context window and the corresponding pinyin appearing in the drug name dictionary. This simple dictionary lookup approach uses the forward maximum match algorithm to search the drug name dictionary (defined in section “drug name dictionaries and lists of relevant terms”).

#### 4.4.3. Feature of Drug Named Entity-Related Terms (F3)

Does the Chinese characters or letters in the current context window match with the terms in the lists related to drug named entities? Also, it uses the forward maximum match algorithm to search term lists (defined in section “drug name dictionaries and lists of relevant terms”).

#### 4.4.4. Feature of Mode (F4)

Other relevant mode features were contained in the *n*-gram texts; for example, does the texts of the current context window contain English letters, numbers, special symbols, or time description?

#### 4.4.5. Feature of Global AN Structure (F5)

F5 indicates whether the name of the section that contains the current *n*-gram texts appears in the predefined section list. One major characteristic of clinical ANs is that the natural language description is context-based. For instance, the medical term “digoxin” in the “Laboratory Results” section is the name of a clinical examination, while the same term in the “Past Medical History” section is the name of a therapeutic drug. We manually reviewed some notes and some articles [38] and defined 15 different section headers (e.g., “History of Illness”).

#### 4.4.6. Feature of Category Annotation (F6)

F6 is the annotation category of the first 3 characters before the current character.

### 4.5. Postprocessing Module

In the annotated narrative text, the annotation “BIO” is resolved as follows: “B” indicates that the character is at the beginning of the drug named entity, “I” shows the character to be in the middle or at the end of the drug named entity, and “O” indicates that the character does not belong to the drug named entity. To guarantee the consistency of character labels and the integrity of name recognition, we also used some simple heuristic rules (see Table 5).

## 5. Experiments

### 5.1. Baseline Systems

First, we constructed baseline system 1, which used the maximum matching algorithm [39] and professional drug dictionaries. It is the most mature and commonly used MER method in China. We then added the maximum matching between terms of drug names and the Chinese pinyin. Based on WM dictionaries and TCM dictionaries, we extracted the drug names from the segmented texts. Like the rule-based term-matching system, which extracts names of genes and proteins from scientific literatures, this baseline system also recognizes named entities by using terminological dictionaries, facilitating the subsequent IE or IR. Since we focus on the performance of ML-based combined systems, this baseline system adopts a preprocessing step like in the main system. Then, we used a simplified CCMER adopting simple local feature set combination F1-1 as baseline system 2 and compared the system performances under different feature sets. Finally, we used a simplified CCMER (excluding the filtration module for classifications of potential hot sentence) adopting full feature set combination (F1 + F3 + F4 + F5 + F6) as baseline system 3 and compared it with the performances of the full-version CCMER system under the same feature set.

### 5.2. Experiment Evaluation Methods

Regarding the characteristics and difficulty in MER recognition in TCM-WM combined Chinese ANs, we used 1 type of soft evaluation indicators [40] that are widely applied in research on Chinese named entities and based the evaluation on standard precision, recall, and F-measure [41]. The main idea is that we score-recognized MERs from three aspects: detection, classification, and boundary. The detailed rules are listed in Table 6.

It should be noted that in clinical practice, “静/B-WM 滴/I-WM 恩/I-WM 度/I-WM 复/O 查/O 有/O 进/O 展/O” is more reliable than “静/O 滴/O 恩/B-WM 度/I-WM 复/I-WM 查/I-WM 有/O 进/O 展/O,” so we assign more scores at the start position of ME.

(4) $$\begin{array}{c} Precision=\frac{soft\;scores\;of\;correctly\;recognized\;MNEs}{number\;of\;recognized\;MNEs};\\ Recall=\frac{soft\;scores\;of\;correctly\;recognized\;MNEs\;}{number\;of\;MNEs\;in\;answer};\\ F‐measure=\frac{2\times recall\times precision}{recall+precision}. \end{array}$$

## 6. Results

### 6.1. Results from Baseline System 1

We first tested the performance of baseline system 1. As shown in Table 7, the overall performance is obviously low, and the F-measures of TCM drug name recognition and WM drug name recognition are both less than 55%. After preliminary analysis, we hypothesized that the performance can be further enhanced if the scale of professional drug name dictionaries was improved.

### 6.2. Results from CCMER

The confusion matrix in Table 8 shows that the test set from the filtration module of potential hot-sentence classification based on SVM uses the sentence classification results, determined from the predefined feature set (defined in “Filtration Module for Classifications of Potential Hot Sentence” section). Clearly, most sentences containing drug name(s) were correctly classified and transferred to the subsequent recognition module for identifying drug named entities, fitting into the accurate, comprehensive, and automatic annotations (i.e., categories) of drug names.

**Figure 2:**
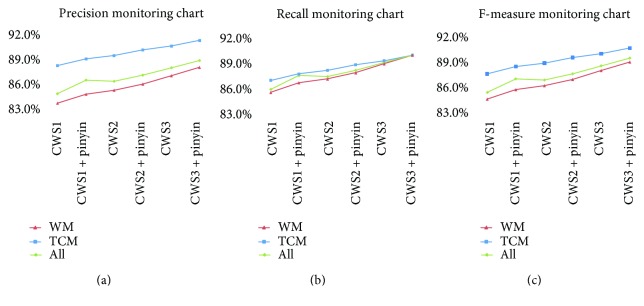
Precision, recall, and F-measure obtained by CRF with different features under different CWS settings.

Figure 2 shows the performance of the CRF-based MER system in terms of *n*-gram local context feature set of Chinese characters and pinyin. The feature set was tested with the candidate hot-sentence subset from the training set. Clearly, the local context feature set helped to improve the performance of MER. In most cases, the use of a larger number of features yielded a higher recognition performance. We compared the system performances between CWS = 3 and CWS = 1. The F-measure of drug named entity recognition using only the feature set of Chinese characters was improved only by 3.1%, while that using the local feature sets of both Chinese characters and pinyin was improved by 4%. Thus, in the subsequent tests, F1-3 + F1-6 (CWS = 3) was used as a feature set (F1) under local text context.

**Figure 3:**
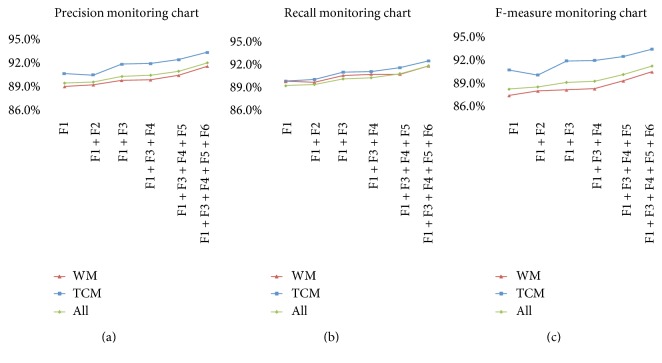
Precision, recall, and F-measure obtained by CRF with different features under different CWS = 3 setting.

With various feature sets, the performances of the CRF-based MER system with the candidate hot-sentence subset are shown in Figure 3. Clearly, the optimal performance is found with F1 + F3 + F4 + F5 + F6. Compared with the only use of F1, the WM drug named entity recognition of ANs has F-measure = 91.2%, while TCM drug named entity recognition of ANs has F-measure = 93.5%, with an increase of 2.5% and 2.7%, respectively. The overall drug named entity recognition has a 2.6% higher F-measure.

**Figure 4:**
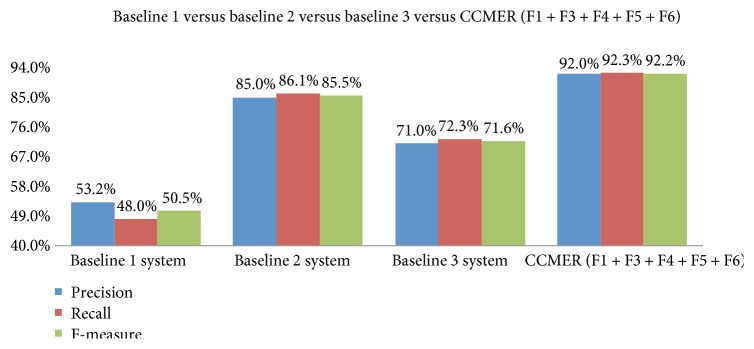
Precision, recall, and F-measure obtained by the baseline systems versus the CCMER system.

The new approach obviously outperforms baseline system 1 based on professional drug dictionaries. As shown in Figure 4, the F-measures of TCM and WM drug named entity recognitions are increased by 45.8% and 38.2%, respectively. Since the filtration module of potential hot-sentence classification also deletes abundant irrelevant sentences, the operation efficiency of the system is largely improved and the operation time is shortened.

Moreover, we also conducted experiments, built baseline system 3 (see Table 9), and determined the contribution of the filtration module of potential hot-sentence classification to CCMER performance. The detection value of hot sentences for CCMER can be seen by comparing the results of baseline system 3 with those of full-version CCMER; the only difference between these two systems is the use of hot-sentence detection. The difference in F-measure of the two systems is as large as 20.6%, with the full-version CCMER achieving an F-measure of 92.2% while baseline system 3 without the filtration module for classifications of potential hot sentence having an F-measure of only 71.6% (Figure 4).

## 7. Discussion

Here, we manually built a dataset involving 972 annotated ANs containing TCM-WM combined treatment. Based on this, we tested a new approach, CCMER, and investigated its performance under different feature allocations. The performance of CCMER is significantly improved versus that of the baseline system 1, as the F-measures of TCM and WM drug named entity recognitions are increased by 45.8% and 38.2%, respectively. The deletion of abundant irrelevant sentences from the dataset results in largely improved operation efficiency.

The optimal performance occurs with the use of a feature set (F1 + F3 + F4 + F5 + F6), as the F-measure of overall drug name recognition is 41.9% higher than that using the baseline system. This indicates that the feature sets with different dimensions are modestly complementary and also proves that the results from Meystre et al. [6] can also be applied to the processing of ANs written in logographic languages.

We then preliminarily studied the contributions of single features to the drug named entity annotation. First, the use of small-scale medical drug name dictionaries (F2) does not improve the system performance. This is not surprising because the same type of information was already captured by F1 and certain drug name entries in F2 lacked comprehensive and detailed information about the drugs. Unfortunately, our self-compiled drug name dictionaries are of small scales. The system performance can be further improved if foreign resources such as Chinese version RxNorm [42] can be combined. In the future, we will refer to Xu et al.' approach [43] to enrich and improve Chinese drug dictionaries. Moreover, F5 helps to reduce the false-positive (FP) rate. For instance, though results of blood drug concentrations, such as “digoxin 0.7 ng/ml” in the “LAB Results” section in ANs, are similar to those of the form of conventional medical orders on medication, the system can automatically delete such results if originated from the section of “LAB Results,” because the system has learned from the training set that there are no drug names for treatment under the contexts of “LAB Results” section. However, false positive still exists in the system. The section of medical orders contains not only orders on drugs but also other contents such as test items. Some of the test items may be presented as drug names such as “folic acid + vitamin B12” and can be mistakenly recognized as drug named entities. Such false-positive results can make drug NER more complicated. Error analysis of the result set shows that F6 helps to determine the end position of drug names, rather than finding out new drug names. However, the final result set contains most characters corresponding to label “O,” so this is also our intuitive evaluation.

Moreover, we find hot-sentence detection in AN texts to be a key factor affecting the systemic performance. The hot-sentence detection technique was a way to determine the focus areas of the texts and thus filter out a large amount of noise. Removing the filtration module for classifications of potential hot sentence alone would largely reduce the systemic performance.

Meanwhile, we found during TCM drug name recognition that TCM is subdivided into Chinese medicinal herbs and Chinese patent drugs. The names of Chinese medicinal herbs are usually composed of 2 to 3 (mean 2.57) Chinese characters, while Chinese patent drugs are preparations made from TCM materials through modern pharmaceutical approach/process complying with quality standards. Their named entities combine the characteristics of both Chinese medicinal herbs and WM; thus, the recognition rates of these drug names are very low. For instance, for Heartleaf *Houttuynia* injection liquid, a Chinese patent drug, “Heartleaf *Houttuynia*” was recognized as “traditional Chinese medicine,” while “injection liquid” was recognized as “Western medicine”; other examples include Radix Salvia miltiorrhizae tablets. Generally, however, the boundaries of TCM drug names are more unidentifiable than those of WM drug names.

### 7.1. Error Sources

Among the annotated results with scores of a soft evaluation indicator equal to zero for medical entities, the major error source is the recognition of general terms of drugs, such as anticoagulants, antibiotics, compound vitamins, and antihypertensive drugs. These general terms were included into the gold standard here, because while drug named entities are important drug use events that are captured by the annotator, the general terms of these drugs might also indicate such important event, yet owing to a lack of support from fine-grained information sources and medical knowledge, the current system cannot recognize them. This is also one research direction in the future.

Another common error occurs only in the sample recognition with the test set, but not with the training set. The supervised ML system has one advantage that it can accurately capture drug names in the test dataset that are not in the training set. This robustness is attributed to the systematic ability of capturing context information. As discussed above, though we annotated 648 ANs as a training set, the annotated dataset at this scale still cannot fully cover the test set. For instance, this system detects “Amoxy” from “Amoxy (Amoxicillin) 0.5 g PO TID” as a drug name, though this drug name is not in the training set. We think that the system learns from the training set through a context mode “<drug name><dosage><drug use approach><frequency>.” On the other hand, the system cannot detect “Amoxy tests negative,” because this context mode does not appear in the training set.

Owing to the timeliness and urgency of clinical work, doctors usually abbreviate and shorthand some drug names, such that “vitamin A and vitamin C” is often abbreviated as “vitamin A, C.” These two drug names share the common beginning characters “维生素 (vitamin),” and the combination of the two drug names is abbreviated to a new simple name combination. Such abbreviated drug descriptions omitting the same beginning or ending characters do not contain “and” or “or”; thus, unlike processing compound descriptions in general texts, in these abbreviated descriptions, recognizing the common beginning or ending characters of the compound-drug names can only result in the correct recognition of the first or last drug name in the combination, while all the other drug names are ignored.

Moreover, standard clinical guidance of treatment based on diagnosis has not been extensively followed in the medical institution where our samples were collected. Doctors in this institution prescribe medication according to previous experiences for most diseases. Thus, for the same disease with the same symptoms, doctors may prescribe different drugs, leading to low appearing frequency of single-drug named entities. Take transfusion medicines as an example, except for solvents like glucose infusion and normal saline solutions; about 50.3% of the medical solvents that are used for transfusion only appear once, which is one cause for the low recognition rates of WM drug names.

### 7.2. Limits of Application

Our approach also has some limitations. First, we only tested the ANs from one data source with one pattern from one medical center. Though CEHRTG is an HL7 CDA R2-based [44] generalized EHR interoperable AN document frame, MERs are also correlated with the ways that ANs are written, habits, and quality of clinicians. Thus, the versatility of this approach in other medical institutions or other types of ANs should be validated [45]. Second, most features used here are only at the level of Chinese characters; using features at the level of semantics is beyond our study. Thus, common problems in clinical ANs, such as coreference resolution and subject main-body examination, cannot be well solved at present. Third, the drug name dictionaries built here are incomplete and of small scale and do not contribute much to the system performance. In the future, we will try other machinery learning techniques feasible for MER, such as supervised ML algorithms (e.g., the hot SSVM), and gradually expand the experimental scope and the types of ANs. To improve the systemic effectiveness and accuracy, we should consider the inclusion of semantic features.

## 8. Conclusions

Here, we targeted at analyzing texts written in Chinese, a typical logographic language; tried MER in nonstructural texts regarding TCM-WM combined treatment; and proposed a new cascade-type approach—CCMER. This approach avoids the side effects due to abundant negative samples and improves the recognition performance of drug named entities in the logographic (Chinese) descriptions. We think that this approach may provide some reference values for MLP of other logographic languages. We also conducted many fine experiments. We found that the *n*-gram information and section information based on Chinese characters and pinyin help to improve the performance of MER. However, the contribution of small-scale professional dictionaries is small. The MER system with the optimal performance was found in the test set involving 324 manually annotated ANs with TCM-WM combined treatment. In this system, the F-measures of TCM and WM drug named entity recognitions are 93.5% and 91.7%, respectively, significantly higher than those in the baseline system.

## Figures and Tables

**Table 1:** Drug name entities and definitions of medication events.

Semantic type	Definition
Drug name entity	Names of TCM or WM drugs used in clinical treatment, including single drugs or drug combinations, general names of drugs, and TCM-specific decoctions and paste formula
Name of WM drug	Drugs used in WM, including chemical name, trade name, common name, and prescription name
Dose of WM	Dosage of WM for each patient each time as per doctor's advice
WM use method	Methods to use WM by a patient as per the doctor's advice
WM use frequency	Time interval for use of a single dose of WM as per the doctor's advice
Name of TCM drug	Different types of TCM products made from TCM materials or with TCM materials as raw materials, in TCD or TCM-WM combined treatment
Dose of TCM	A TCM-exclusive concept, including 3 cases: potions, tablets, and pills. Weight of TCM potions and number of TCM tablets or pills following the doctor's advice
TCM drug form	A TCM-exclusive concept or a TCM application mode in adaptation to requirements of treatment or prevention following the doctor's advice
TCM overall dose	A TCM-exclusive concept or the total number of medications following the doctor's advice
TCM use frequency	Time interval for use of a single dose of TCM following the doctor's advice
TCM use method	Methods to use TCM by a patient following the doctor's advice
TCM use requirements	A TCM-exclusive concept or the conditions met by a patient to use TCM following the doctor's advice

**Table 2:** Name annotation rules of core drugs.

Number	Rule description
1	Drug information should be recorded in the ANs, including the names of disease-treating or symptom-relieving drugs (e.g., TCM, WM, and biological agents). Drug name defined here describes one drug, one drug combination, or one medical product
2	The modifiers indicating a change of drug use or a patient's drug use duration should not be included in the annotated drug name
3	Drug name entity is annotated by 1 phrase. For instance, the drug “nifedipine GITS” is usually annotated by two phrases: nifedipine and GITS, while here we annotate the whole drug name phrase as one drug name entity or namely the whole entity should be ascribed as one phrase
4	For TCM, Chinese characters indicating drug forms, such as “丸” (pill), “粉” (powder), and “汤” (decoction), cannot be annotated as single characters, because they are usually placed as the last characters within certain TCM drug names, and thus, the drug names should be annotated as a whole. For example, in the TCM drug name “大青龙汤” (da qin long decoction), “da qin long” is the pinyin to Chinese characters “大青龙,” while Chinese character “汤” is a drug form meaning “decoction”
5	The explicit negative modifiers around the drug names are not included in the annotated drug name entity
6	When Chinese drug name and the corresponding English name coexist in one short description without other words between them, they are jointly annotated as one drug name entity
7	When Chinese drug name and the corresponding English name coexist in one short description with simple symbols such as “/” or “-” between them, they are jointly annotated as one drug name entity
8	We also have seen parallel construction or ellipsis construction in some drug names. If two drug names are connected by one conjunction, the two drug names should be annotated as two separate drug entities
9	In some situations, certain words and punctuations in a drug name entity are ignored. Then, the following rules are used: (a) Conjunctive words and “、” and “,” are ignored and excluded in drug name entities (b) To reserve the functionality and readability of drug name entities, we should annotate these descriptions as a separation of two independent drug name entities
10	If two or more valid drug names end with the same characters and are combined together, then, the last drug name with the ending characters is taken as one complete drug name. For instance, in a description of two drug names ofloxacin and vitamin C injection, vitamin C injection is recognized as one complete drug name entity
11	Drug names usually contain figures, letters, and other symbols. Since these symbols represent drug-related information (e.g., (), <>), they are included in one drug name entity
12	When the TCM name and the description of the producing area coexist in the drug name, the information of the producing area is ignored. For instance, in Chuan Bei Mu (Zhejiang), Zhejiang is ignored
13	Specification may follow a drug name that does not belong to a drug name entity and may not need separate annotation. For example, in the drug name Cold Clear Capsule (a capsule with 24 mg of paracetamol), the specification is in brackets
14	Maximum annotation length of drug name entities should be set and followed, except when such a limitation of annotation length destroys the validity of the grammar structure. Especially, when modifiers of a drug name contain special information about a brand and pattern and form an agglutinate structure within the drug name, then, these modifiers should be included in the drug name entity. For example, in the drug name “苗泰小儿柴桂退烧颗粒” (pinyin translation is “miao tai xiao er chai gui tui sao ke li”), “苗泰” (pinyin: “miao tai”) is a drug brand and should not be excluded

**Table 3:** Dataset scales used in this study.

Dataset name	Number of ANs	Number of sentences	Number of sentences mentioning drug name	Number of annotated WM drug name entities	Number of annotated TCM drug name entities	Number of annotated drug name entities
Training set	648	40,649	1665	1322	487	1809
Test set	324	20,397	716	581	209	790
Total	972	61,046	2381	1903	696	2599

**Table 4:** List of various features for the drug name recognizer.

Feature set	Features	Description
F1-1	CWS = 1: 1‐gram = C_*i*−1_, C_*i*_, C_*i*+1_ 2‐gram = C_*i*−1_ C_*i*_, C_*i*_C_*i*+1_ 3‐gram = C_*i*−1_ C_*i*_C_*i*+1_	The 1-gram, 2-gram, and 3-gram of the character text at CWS = 1
F1-2	CWS = 2: 1‐gram = C_*i*−2_, C_*i*−1_, C_*i*_, C_*i*+1_, C_*i*+2_ 2‐gram = C_*i*−2_ C_*i*−1_, C_*i*−1_ C_*i*_, C_*i*_C_*i*+1_, C_*i*+1_C_*i*+2_ 3‐gram = C_*i*−2_ C_*i*−1_C_*i*_, C_*i*−1_ C_*i*_C_*i*+1_, C_*i*+1_ C_*i*+2_C_*i*+3_	The 1-gram, 2-gram, and 3-gram of the character text at CWS = 2
F1-3	CWS = 3: 1‐gram = C_*i*−3_, C_*i*−2_, C_*i*−1_, C_*i*_, C_*i*+1_, C_*i*+2_, C_*i*+3_ 2‐gram = C_*i*−3_ C_*i*−2_, C_*i*−2_ C_*i*−1_, C_*i*−1_ C_*i*_, C_*i*_C_*i*+1_, C_*i*+1_C_*i*+2_, C_*i*+2_C_*i*+3_ 3‐gram = C_*i*−3_ C_*i*−2_C_*i*−1_, C_*i*−2_ C_*i*−1_C_*i*_, C_*i*−1_ C_*i*_C_*i*+1_, C_*i*+1_ C_*i*+2_C_*i*+3_, C_*i*+2_ C_*i*+3_C_*i*+4_	The 1-gram, 2-gram, and 3-gram of the character text at CWS = 3
F1-4	CWS = 1: 1‐gram = P_*i*−1_, P_*i*_, P_*i*+1_ 2‐gram = P_*i*−1_ P_*i*_, P_*i*_P_*i*+1_ 3‐gram = P_*i*−1_ P_*i*_P_*i*+1_	The 1-gram, 2-gram, and 3-gram of the pinyin corresponding to the current character at CWS = 1
F1-5	CWS = 2: 1‐gram = P_*i*−2_, P_*i*−1_, P_*i*_, P_*i*+1_, P_*i*+2_ 2‐gram = P_*i*−2_ P_*i*−1_, P_*i*−1_ P_*i*_, P_*i*_P_*i*+1_, P_*i*+1_P_*i*+2_ 3‐gram = P_*i*−2_ P_*i*−1_P_*i*_, P_*i*−1_ P_*i*_P_*i*+1_, P_*i*+1_ P_*i*+2_P_*i*+3_	The 1-gram, 2-gram, and 3-gram of the pinyin corresponding to the current character at CWS = 2
F1-6	CWS = 3: 1‐gram = P_*i*−3_, P_*i*−2_, P_*i*−1_, P_*i*_, P_*i*+1_, P_*i*+2_, P_*i*+3_ 2‐gram = P_*i*−3_ P_*i*−2_, P_*i*−2_ P_*i*−1_, P_*i*−1_ P_*i*_, P_*i*_P_*i*+1_, P_*i*+1_P_*i*+2_, P_*i*+2_P_*i*+3_ 3‐gram = P_*i*−3_ P_*i*−2_P_*i*−1_, P_*i*−2_ P_*i*−1_P_*i*_, P_*i*−1_ P_*i*_P_*i*+1_, P_*i*+1_ P_*i*+2_P_*i*+3_, P_*i*+2_ P_*i*+3_P_*i*+4_	The 1-gram, 2-gram, and 3-gram of the pinyin corresponding to the current character at CWS = 3
F2-1	InDictTCM	Are the current character and the surrounding characters contained in the TCM dictionary?
F2-2	InDictTCMPinyin	Are the pinyins corresponding to the current character and the surrounding characters contained in the TCM dictionary?
F2-3	InDictWM	Are the current character and the surrounding characters contained in the WM dictionary?
F2-4	InDictWMPinyin	Are the pinyins corresponding to the current character and the surrounding characters contained in the WM dictionary?
F3-1	CurC_*x*_-HasTCMDoseUnit	Do the current character and subsequent characters contain the TCM dosage unit *x* = {*i*, *i* + 1, *i* + 2, *i* + 3} at CWS = 3?
F3-2	CurC_*x*_-HasWMDoseUnit	Do the current character and subsequent characters contain the WM dosage unit *x* = {*i*, *i* + 1, *i* + 2, *i* + 3} at CWS = 3?
F3-3	PreC_*x*_-HasTCMDoseUnit	Do the characters before the current character contain the TCM dosage unit *x* = {*i* − 1, *i* − 2, *i* − 3} at CWS = 3?
F3-4	PreC_*x*_-HasWMDoseUnit	Do the characters before the current character contain the WM dosage unit *x* = {*i* − 1, *i* − 2, *i* − 3} at CWS = 3?
F3-5	CurC_*x*_-HasTCMRoute	Do the current character and subsequent characters contain the TCM usage term *x* = {i, *i* + 1, *i* + 2, *i* + 3} at CWS = 3?
F3-6	CurC_*x*_-HasWMRoute	Do the current character and subsequent characters contain the WM usage term *x* = {*i*, *i* + 1, *i* + 2, *i* + 3} at CWS = 3?
F3-7	PreC_*x*_-HasTCMRoute	Do the characters before the current character contain the TCM usage term *x* = {*i* − 1, *i* − 2, *i* − 3} at CWS = 3?
F3-6	PreC_*x*_-HasWMRoute	Do the characters before the current character contain the WM usage term *x* = {*i* − 1, *i* − 2, *i* − 3} at CWS = 3?
F3-9	CurC_*x*_-HasTCMFormUnit	Do the current character and subsequent characters contain the TCM drug form unit *x* = {*i*, *i* + 1, *i* + 2, *i* + 3} at CWS = 3?
F3-10	CurC_*x*_-HasWMFormUnit	Do the current character and subsequent characters contain the WM drug form unit *x* = {*i*, *i* + 1, *i* + 2, *i* + 3} at CWS = 3?
F3-11	PreC_*x*_-HasTCMFormUnit	Do the characters before the current character contain the TCM drug form unit *x* = {*i* − 1, *i* − 2, *i* − 3} at CWS = 3?
F3-12	PreC_*x*_-HasWMFormUnit	Do the characters before the current character contain the WM drug form unit *x* = {*i* − 1, *i* − 2, *i* − 3} at CWS = 3?
F3-13	CurC_*x*_-HasTCMFrequency	Do the current character and subsequent characters contain the TCM frequency description *x* = {*i*, *i* + 1, *i* + 2, *i* + 3} at CWS = 3?
F3-14	CurC_*x*_-HasWMFrequency	Do the current character and subsequent characters contain the WM frequency description *x* = {*i*, *i* + 1, *i* + 2, *i* + 3} at CWS = 3?
F3-15	PreC_*x*_-HasTCMFrequency	Do the characters before the current character contain the TCM frequency description *x* = {*i* − 1, *i* − 2, *i* − 3} at CWS = 3?
F3-16	PreC_*x*_-HasWMFrequency	Do the characters before the current character contain the WM frequency description *x* = {*i* − 1, *i* − 2, *i* − 3} at CWS = 3?
F4-1	HasNum9	Do the current character and the surrounding characters include the figure “9”?
F4-2	HasToken@	Do the current character and the surrounding characters include the symbol “@”?
F4-3	HasEnglishAlphabets	Do the current character and the surrounding characters include English letters?
F4-4	HasTime	Do the current character and the surrounding characters contain time description such as hour, week, date, or year?
F5	InListSectionName	Do the name of AN section involving the current character and the surrounding characters appear in the predefined section list?
F6	Class_*x*_ = [BIO]	These three types of features indicate the type labels of the 3 characters before the current character *x* = {*i* − 1, *i* − 2, *i* − 3}

**Table 5:** Rules used in the postprocessing module.

Number	Description of postprocessing rules
1	If the label “O” is followed by the label “I,” then, “I” is forcefully resolved to the same-type label “B”
2	If “B” is followed by a different-type label “I,” then, “I” is forcefully resolved to “B,” such as B-WM I-TCM ➔ B-WM B-WM
3	In Chinese ANs, the end of a drug name is rarely followed by another completely different therapeutic drug. In this case, we established the following rules, such as B-WM I-WM B-WM I-WM ➔ B-WM I-WM I-WM I-WM
4	If a drug name entity only contains “),” but not “(,” the starting position of the current drug name entity is moved ahead, while the label “B” is repositioned at the position of “(”
5	If “)” is annotated as label “O” and it immediately follows the end of the Chinese characters of the recognized drug name, then, this field end is expanded by one character to involve “)”; otherwise, the starting position of the field of the drug name is adjusted to be discarded “(”

**Table 6:** Rules used in evaluation.

Score	Rule description
1	Medication entity is accurately detected, and divisions of class and boundary are both correct
0.8	Only one error is detected at the start position of the ME boundary
0.6	Only one error is detected at the end position of the ME boundary
0.4	Two errors are detected at the start and end positions of ME boundaries, respectively
0	ME is not detected, or the detected phrase is not a drug name entity annotated in the gold standard

**Table 7:** Performance of the baseline system 1 based on professional drug dictionaries and the maximum matching algorithm between drug name characters and pinyin.

	Precision	Recall	F-measure
TCM drug name	49.2%	45.5%	47.3%
WM drug name	54.9%	49.1%	51.8%
All drug names	53.2%	48.0%	50.5%

Note: to the nearest 0.1%.

**Table 8:** Confusion matrix of outputs from the filtration module of potential hot-sentence classification.

Classification	Medication	No medication	Total
Medication	**695**	21	716
No medication	57	**19,624**	19,681
Total	752	19,645	20,397

**Table 9:** Performance of the baseline system 3 based on CCMER (not on the use of hot-sentence detection) (feature sets: F1 + F3 + F4 + F5 + F6).

	Precision	Recall	F-measure
TCM drug name	73.4%	71.3%	72.3%
WM drug name	70.2%	72.3%	71.4%
All drug names	71.0%	72.3%	71.6%

Note: to the nearest 0.1%.

## Abbreviations

MER: Medication entity recognition
AN: Admission note
TCM: Traditional Chinese medicine
WM: Western medicine
CCMER: A cascade-type Chinese medication entity recognizer
SVM: Support vector machine
CRF: Conditional random field
I2B2: Informatics for Integrating Biology and the Bedside
NTCIR: NII Testbeds and Community for Information access Research
NER: Named entity recognition
EHR: Electronic health record
CEHRTG: Chinese Electronic Medical Record Template Guide
MLP: Medical language processing
ML: Machine learning
IR: Information retrieval
SAHZU: Second Affiliated Hospital Zhejiang University School of Medicine
NLP: Natural language processing
IAA: Interannotator agreement
CWS: Context window size
IE: Information extraction
WF: Word feature
FP: False positive
SF1: In symbol list
SF2: In Chinese drug name dictionaries
SF3: In pinyin dictionaries
SF4: Positive set of statistical word feature
SF5: Negative set of statistical word features
SF6: In event patterns
F1: The local context feature
F2: Feature of drug name dictionary
F3: Feature of drug name entity-related terms
F4: Feature of mode
F5: Feature of global AN structure
F6: Feature of category annotation.
